# Introducing Pitt-Hopkins syndrome-associated mutations of *TCF4* to *Drosophila daughterless*

**DOI:** 10.1242/bio.014696

**Published:** 2015-11-30

**Authors:** Laura Tamberg, Mari Sepp, Tõnis Timmusk, Mari Palgi

**Affiliations:** Laboratory of Molecular Neurobiology, Department of Gene Technology, Tallinn University of Technology, Akadeemia Rd.15, Tallinn 12618, Estonia

**Keywords:** Pitt-Hopkins syndrome, *Drosophila melanogaster*, Intellectual disability, Daughterless, bHLH, Nervous system

## Abstract

Pitt-Hopkins syndrome (PTHS) is caused by haploinsufficiency of *Transcription factor 4 (TCF4)*, one of the three human class I basic helix-loop-helix transcription factors called E-proteins. *Drosophila* has a single E-protein, Daughterless (Da), homologous to all three mammalian counterparts. Here we show that human TCF4 can rescue Da deficiency during fruit fly nervous system development. Overexpression of Da or TCF4 specifically in adult flies significantly decreases their survival rates, indicating that these factors are crucial even after development has been completed. We generated *da* transgenic fruit fly strains with corresponding missense mutations R578H, R580W, R582P and A614V found in TCF4 of PTHS patients and studied the impact of these mutations *in vivo*. Overexpression of wild type Da as well as human TCF4 in progenitor tissues induced ectopic sensory bristles and the rough eye phenotype. By contrast, overexpression of Da^R580W^ and Da^R582P^ that disrupt DNA binding reduced the number of bristles and induced the rough eye phenotype with partial lack of pigmentation, indicating that these act dominant negatively. Compared to the wild type, Da^R578H^ and Da^A614V^ were less potent in induction of ectopic bristles and the rough eye phenotype, respectively, suggesting that these are hypomorphic. All studied PTHS-associated mutations that we introduced into Da led to similar effects *in vivo* as the same mutations in TCF4 *in vitro*. Consequently, our *Drosophila* models of PTHS are applicable for further studies aiming to unravel the molecular mechanisms of this disorder.

## Abstract (precis)

**Summary:** Introducing mutations of the *TCF4* gene found in human patients into its fly orthologue *daughterless* allows the generation of *Drosophila* models for research into Pitt-Hopkins syndrome.

## INTRODUCTION

Pitt-Hopkins syndrome (PTHS, OMIM #610954) is a rare human disorder characterised by severe developmental delay, autistic behaviours, absence of speech, distinct facial features, epilepsy, constipation and hyperventilation (Pitt and Hopkins, 1978; Whalen et al., 2012). PTHS is caused by haploinsufficiency of the *Transcription factor 4* (*TCF4*, located at 18q21.1, OMIM #602272) (Amiel et al., 2007; Brockschmidt et al., 2007; Zweier et al., 2007). Large chromosomal deletions, partial gene deletions, frame shift, nonsense, splice site or missense mutations in the *TCF4* gene have been found in PTHS patients. These mutations are usually sporadic, but in some cases children have inherited the mutant allele from a mosaic parent (reviewed in Sweatt, 2013). *In vitro,* PTHS-associated missense mutations result in hypomorphic, non-functional or dominant-negative *TCF4* alleles (Sepp et al., 2012). It is unclear whether mutations causing PTHS impair development of the nervous system or functioning of the adult central nervous system (CNS), or both. In addition to PTHS, *TCF4* is associated with several other human diseases such as schizophrenia, Fuchs' corneal endothelial dystrophy and primary sclerosing cholangitis (reviewed by Forrest et al., 2014).

TCF4 (previously also known as ITF2, SEF2 or E2-2) belongs to the family of class I basic helix-loop-helix (bHLH) transcription factors (Massari and Murre, 2000) and should be distinguished from T-cell factor 4 (TCF4/TCF7L2) involved in the Wnt signalling pathway. The bHLH transcription factors form a large evolutionarily conserved family with important roles in numerous developmental processes including neurogenesis, myogenesis, haematopoiesis, and sex determination. The highly conserved bHLH region mediates interaction with other bHLH proteins and specific binding to DNA. Class I bHLH proteins, also called the E-proteins, comprise the mammalian TCF3/E2A, TCF4, TCF12/HEB and the *Drosophila* Daughterless (Da) (Massari and Murre, 2000). They are widely expressed and form homo- or heterodimers with class II bHLH proteins to bind DNA at the Ephrussi box (E-box) sequence, CANNTG. Class II bHLH proteins (Achaete-Scute complex proteins, MyoD, Myogenin, Atonal family etc.) are expressed in a tissue-specific manner but are not capable of activating transcription without E-proteins. In this study, we used the only E-protein in *Drosophila*, Da, to study PTHS-related mutations in the fruit fly.

Being the sole E-protein in *Drosophila*, Da has multiple roles in development – in sex determination and oogenesis (Cronmiller and Cummings, 1993; Smith et al., 2002), neurogenesis (Caudy et al., 1988; Hassan and Vaessin, 1997; Vaessin et al., 1994), eye development (Bhattacharya and Baker, 2011; Lim et al., 2008; Tanaka-Matakatsu et al., 2014), intestine stem cell maintenance (Bardin et al., 2010), and mesoderm development (Castanon et al., 2001). Da can both homodimerise or form heterodimers with class II bHLH proneural proteins and regulate the establishment of neural precursors (Cabrera and Alonso, 1991; Murre et al., 1989; Smith and Cronmiller, 2001; Tanaka-Matakatsu et al., 2014; Troost et al., 2015; Zarifi et al., 2012). Both lack of Da and ubiquitous overexpression of Da result in embryonic lethality, showing the importance of proper dosage (Cronmiller and Cline, 1987; Giebel et al., 1997).

Previously, *Drosophila melanogaster* has been successfully used to model human neurodegenerative diseases (Sang and Jackson, 2005). Recently efforts have been made towards exploiting fruit fly to model neuropsychiatric diseases and intellectual disability disorders (O'Kane, 2011; van der Voet et al., 2014). However, so far there are no fruit fly models of PTHS. Being the only E-protein in *Drosophila,* Da is probably a functional orthologue of all mammalian E-proteins. Here we prove that TCF4 is a true functional orthologue of Da capable of mediating neuronal development in *Drosophila*.

To recapitulate the PTHS in *Drosophila* we introduced four PTHS-associated mutations of TCF4 – R580W, R578H, R582P and A614V – into Da. Their transcriptional activation capability was compared and analysed *in vivo*. All these mutations caused similar defects as their counterparts in TCF4 *in vitro* ranging from hypomorphic to dominant-negative effects.

Finally we show that activation of *da* transgenes with and without PTHS mutations in adult *Drosophila* leads to a reduced lifespan ranging from a few days to couple of weeks. These results implicate that in addition to their roles in development, expression of E-proteins has to be regulated in a spatially and temporally restricted manner also in adult flies.

## RESULTS

### Daughterless is the only orthologue of human E-proteins in *Drosophila*

**Fig. 1.**
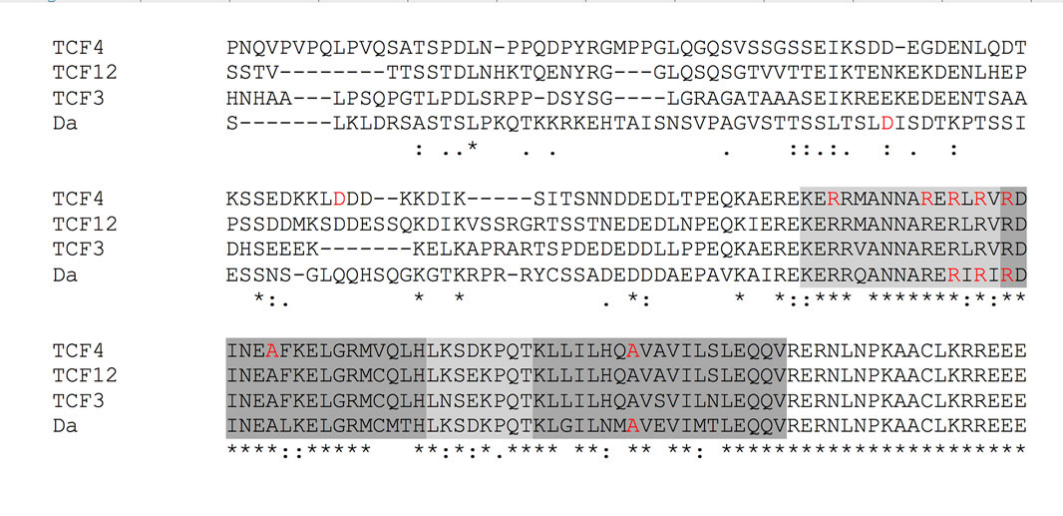
**Human E-proteins and Drosophila Da show high amino acid sequence conservation of bHLH domains.** The amino acid sequences of all three human E-proteins TCF3, TCF4, and TCF12 together with *Drosophila* Da bHLH domains with surrounding sequences are presented. The basic and loop regions are marked as light grey while the helices are coloured dark grey. Amino acid residues marked in red in TCF4 sequence are found mutated in PTHS patients and amino acid residues marked in red in Da sequence where mutations were introduced in this study.

Amino acid sequence analysis showed that Da is about 35% identical to human E-proteins TCF3, TCF4, and TCF12 and has the highest identity with TCF4 (35.54%). Even though the entire amino acid sequence homology between human E-proteins and Da is below 50%, the amino acid identity of bHLH domains between Da and human E-proteins reaches 75% (Fig. 1) which allows extrapolation of mutations found in bHLH of TCF4 of PTHS patients into Da.

Seven out of nine PTHS-associated missense mutations in TCF4 are found in the bHLH region (Sepp et al., 2012). From these bHLH positioned conserved mutations we selected four R578H, R580W, R582P and A614V for introduction into Da (Fig. 1). We named all mutations after PTHS-associated mutations in TCF4, although the numeral positions in Da differ by 14 amino acids (R564H, R566W, R568P, and A600V respectively). Two additional mutations were generated in order to study the importance of amino acid position and specificity. The first of these is D515G (D501G in Da) in nonconserved region close to the mutation D535G found in a PTHS patient. The second is R580L (R566L in Da) where leucine replaces arginine 580 originally found to be mutated into tryptophan in a patient of PTHS. The obtained transgenic fly strains with PTHS mutations introduced to *da* (*da^PTHS^*) were crossed to the *da^G32^*-GAL4 line and verified to produce mutated transcripts (Fig. S1).

### Mutated Daughterless proteins have variable transactivation capabilities in HEK293 cells in comparison to wild type protein

**Fig. 2.**
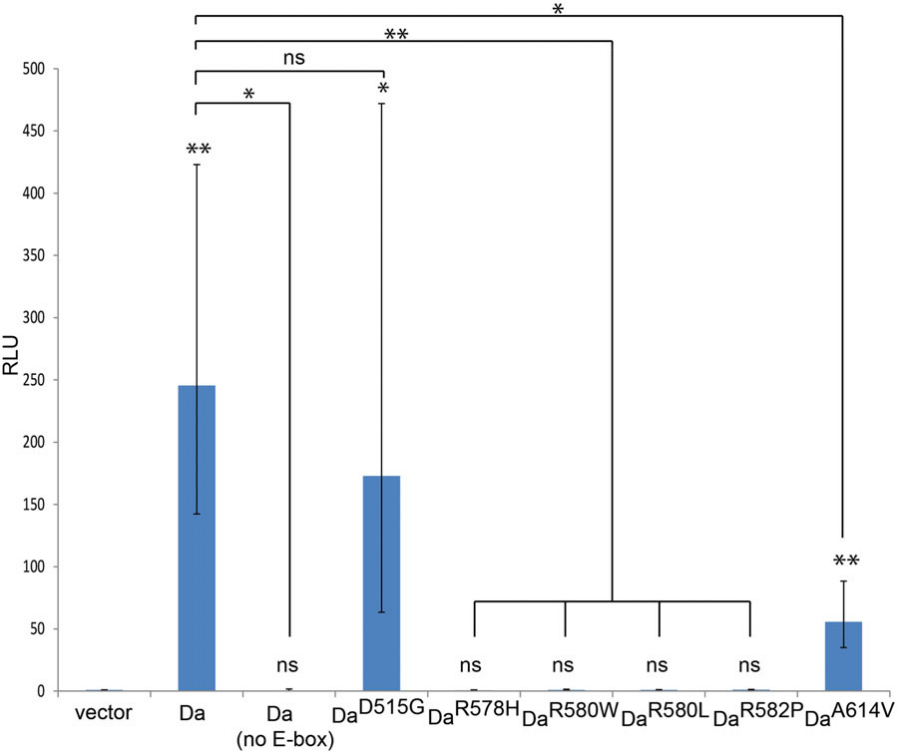
**E-box controlled luciferase reporter transcription activation capabilities of Da^wt^ and Da^PTHS^*in vitro*.** HEK293 cells were co-transfected with wt or mutated *da* construct, firefly luciferase construct carrying 12 µE5 boxes and a minimal promoter, or just a minimal promoter (no E-box), and *Renilla* luciferase construct with minimal promoter for normalisation. Luciferase activities were measured and data are presented as fold induced levels compared to the signals obtained from cells transfected with an empty vector. The mean results from three independent transfection experiments performed in duplicates are shown. Error bars show standard deviations. Statistical significance is shown with asterisks relative to empty-vector transfected cells, or between the groups connected with lines. *P<0.05, **P<0.01, ***P<0.001, ns, not significant, Student *t*-test; RLU, relative luciferase unit.

We and others have previously demonstrated that several PTHS-associated missense mutations impair the functions of TCF4 homodimers and/or heterodimers with ASCL1 *in vitro* (Zweier et al., 2007; de Pontual et al., 2009; Sepp et al., 2012; Forrest et al., 2012). Particularly, PTHS-associated mutations R578H, R580W and R582P abolish the DNA-binding and transactivational capacity of TCF4 homodimers and TCF4:ASCL1 heterodimers, whereas A614V mutation impairs the functions of TCF4 homodimer, but retains the activity of the TCF4:ASCL1 heterodimer (Sepp et al., 2012). To test the transactivation capability of Da proteins carrying the same PTHS-associated mutations and two additional mutations (D515G and R580L), we used luciferase reporter assay in human embryonic kidney-derived cell line (HEK293). Three out of seven constructs tested – Da^wt^, Da^D515G^, and Da^A614V^ – were capable of activating E-box controlled luciferase gene transcription (Fig. 2). Da^wt^ did not activate transcription from the reporter construct without E-boxes indicating that the transcriptional activation is E-box specific. The following mutations abolished reporter gene expression: R578H, R580W, R580L and R582P. In the case of Da^A614V^ the luciferase signal was lower compared to Da^wt^ whereas the control mutation D515G showed no effect on Da transactivation capability. These results indicate that arginines R578 and R580 in the basic region and R582 in the beginning of the first helix of Da bHLH domain are essential for activating E-box controlled transcription*.* The mutation A614V in the second helix shows diminished transactivation of transcription (Fig. 2). The above results are completely consistent with the results obtained with TCF4 proteins carrying the same mutations (Sepp et al., 2012).

### TCF4 and Da, but not Da^R578H^, Da^R580W^, Da^R580L^ or Da^R582P^, activate E-box controlled reporter gene expression in wing disc and induce ectopic thoracic bristles in *Drosophila*

**Fig. 3.**
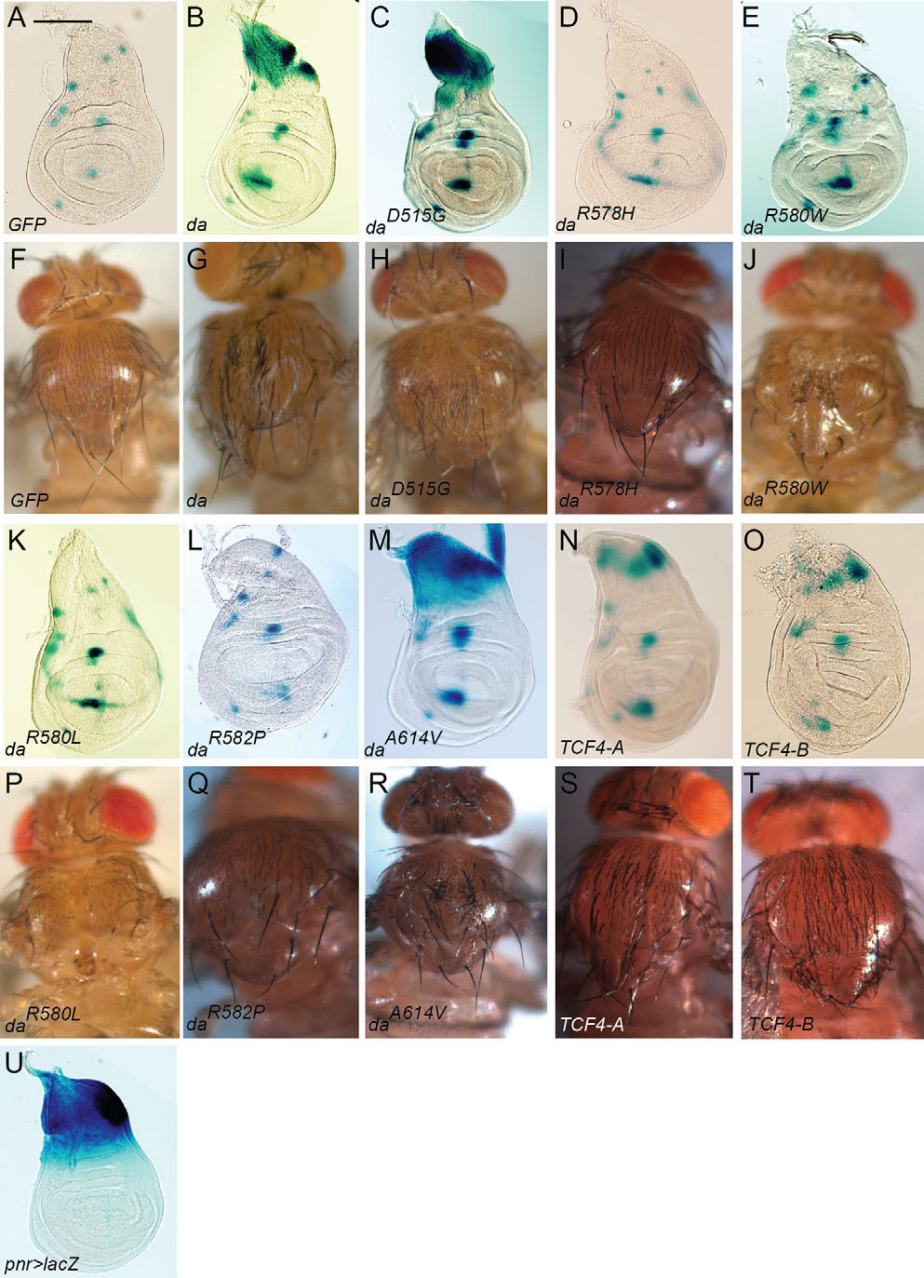
***In vivo E-box-*lacZ reporter assay in larval wing discs and activation of bristle formation by overexpression of Da^wt^, Da^PTHS^, TCF4-A and TCF4-B.** Expression of GFP under *pnr*-GAL4 served as a control in wing disc resembling endogenous activation of *E-box*-lacZ reporter expression (A) and shows no deviation from wild type bristles and thorax (F). Da^wt^ (B), Da^D515G^ (C), Da^A614V^ (M), TCF4-A (N) and TCF4-B (O) were able to activate E-box controlled lacZ expression in *pnr*-GAL4 pattern and caused ectopic bristle formation on adult thorax (G,H,R-T). Da^R578H^ (D), Da^R580W^ (E), Da^R580L^ (K) and Da^R582P^ (L) were unable to activate reporter expression. Da^R580W^ (J), Da^R580L^ (P) and Da^R582P^ (Q) caused thorax malformation, Da^R578H^ (I) had no effect on thorax but induced occasional extra bristles. The expression pattern of *pnr*-GAL4 is shown in blue on panel U. Scale bar on A represents 100 μm and is applicable for A-E,K-O and U.

To analyse the functional consequences of Da mutations *in vivo*, we took the following approaches. First, we analysed the capacity of the overexpressed mutants to activate E-box dependent transcription in wing disc. Second, we tested the impact of the mutations on formation of ectopic sensory bristles induced by Da overexpression (Zarifi et al., 2012). To this end, transgenic *Drosophila* strains were generated expressing Da^PTHS^ under GAL4 control element UAS (Brand and Perrimon, 1993). Additionally, we generated flies with two most widely expressed alternative splice forms of *TCF4* – shorter *TCF4-A* isoform and the longer *TCF4-B* isoform (Sepp et al., 2011). *In vivo* lacZ reporter assay was performed using the transgenic flies with four E-boxes CATCTG upstream of lacZ reporter region as previously described (Culi, Modolell, 1998; Zarifi et al., 2012). UAS-transgenes were ectopically expressed under *pannier*-GAL4 (*pnr-*GAL4) in wing disc notum region (Fig. 3). Notum showed small spot-like areas of transgenic E-box activation by endogenous Da. Expression of GFP was used as a control showing no activation of reporter lacZ and mimicking transgenic E-box activation by endogenous Da (Fig. 3A). Importantly, this control did not induce ectopic bristles or thorax defects (Fig. 3F). Da^wt^ (Fig. 3B), Da^D515G^ (Fig. 3C), Da^A614V^ (Fig. 3M), and to a lesser extent TCF4-A (Fig. 3N) and TCF4-B (Fig. 3O) were able to activate reporter transcription from the E-box *in vivo*. Additionally, the same transgenes induced ectopic bristle formation on thorax (Fig. 3G,H,R-T). Arginine mutations R578H (Fig. 3D), R580W (Fig. 3E), R580L (Fig. 3K) and R582P (Fig. 3L) abolished the ability of Da to activate reporter expression in the wing disc. Interestingly, Da^R580W^ (Fig. 3J), Da^R580L^ (Fig. 3P) and Da^R582P^ (Fig. 3Q) reduced the number and size of thoracic bristles and caused malformation of the whole adult thorax indicating that these mutations share dominant-negative effects. Overexpression of Da^R578H^ (Fig. 3I) resulted in no major defects on the thorax, but on several occasions formation of some ectopic bristles was observed. Taken together, these results demonstrate that TCF4 isoforms TCF4-A and TCF4-B, Da^wt^, Da^D515G^ and Da^A614V^ activate E-box controlled transcription in *Drosophila*. The arginine mutations R580W, R580L and R582P cause dominant negative effects while the mutation R578H considerably reduces the ability of Da to induce ectopic bristles.

### Da, Da^D515G^, Da^R578H^, Da^A614V^, TCF4-A and TCF4-B are capable of rescuing *da* null embryonic neuronal phenotype

Next we asked whether Da^wt^ or any of the mutants used is able to rescue *da* null lethality or severe embryonic nervous system phenotype with total lack of peripheral nervous system (PNS) and disrupted CNS (Caudy et al., 1988). For this experiment transgenic fruit fly strains expressing Da^wt^, Da^PTHS^, TCF4-A or TCF4-B under GAL4 responsive element were crossed to the GAL4 lines in *da* null background. First, we performed the rescue experiments using nervous system specific *GMR12B08*-GAL4 made of the only intron of *da* gene fused with *Drosophila* core synthetic promoter followed by GAL4 coding region (Pfeiffer et al., 2008). *GMR12B08*-GAL4 drives expression specifically in the nervous system in all developmental stages. This driver failed to rescue *da* null embryonic lethality with all our transgenes. The result obtained is consistent with known functions of Da outside the nervous system, for example in the mesoderm and muscle development (Castanon et al., 2001; Gonzalez-Crespo and Levine, 1993; Wong et al., 2008).

**Fig. 4.**
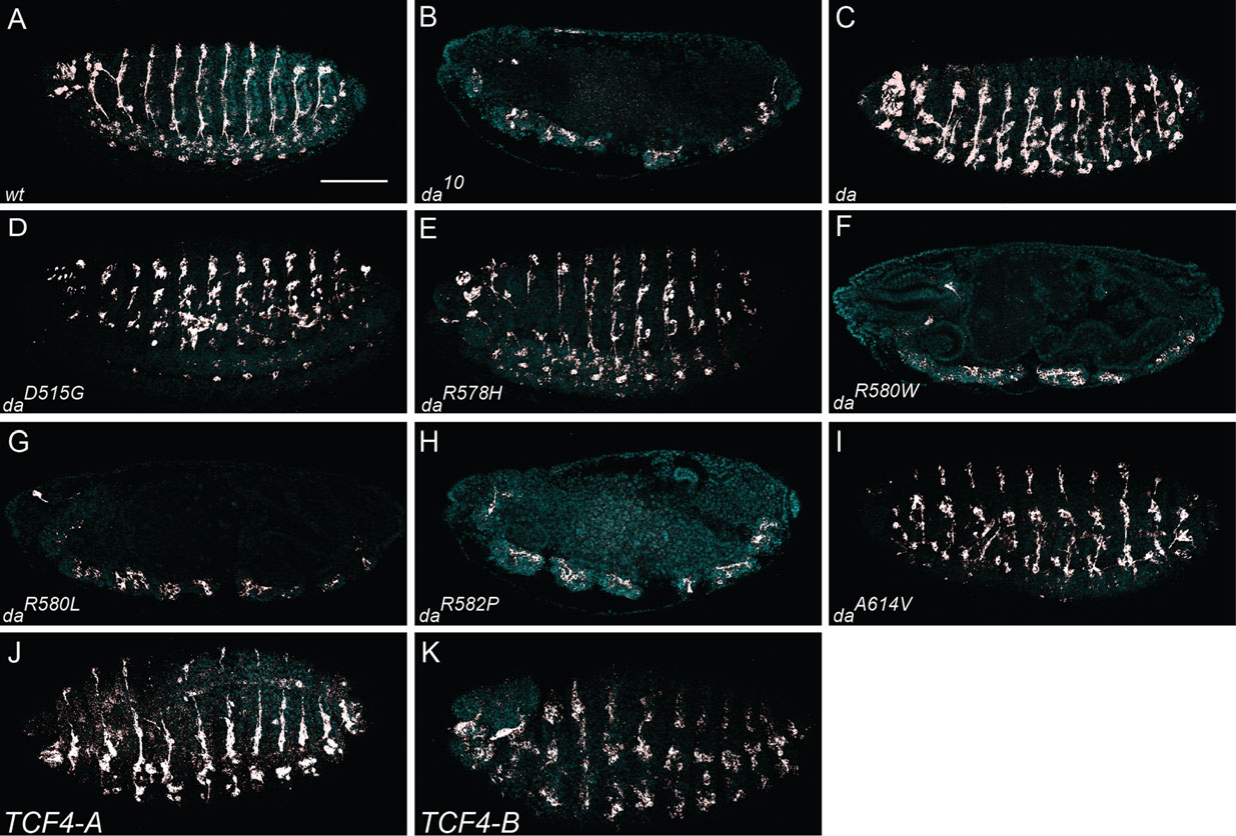
**Rescue of *da* null embryonic neuronal phenotype by expressing Da^wt^, Da^PTHS^, TCF4-A or TCF4-B under *da^G32^*-GAL4.** Neuronal marker Futsch expression is shown in white. DAPI, in light blue, marks nuclei. Compared to *wt* embryos (A), homozygous *da^10^* embryos lack PNS and CNS is discontinuous (B). Overexpression of Da^wt^ (C), Da^D515G^ (D), Da^R578H^ (E), Da^A614V^ (I), TCF4-A (J) and TCF4-B (K) under *da^G32^-*GAL4 rescued peripheral nervous system. All three arginine mutations Da^R580W^ (F), Da^R580L^ (G) and Da^R582P^ (H) failed to rescue *da* null nervous system phenotype as the CNS is still fragmented and PNS missing. Anterior side is to the left and dorsal side is up. Scale bar represents 100 μm.

Subsequently, we repeated the rescue experiment with Da^wt^ under ubiquitous *da^G32^*-GAL4 in *da* null background that led to embryonic lethality (Giebel et al., 1997; Smith and Cronmiller, 2001). Despite embryonic lethality, the severe nervous system phenotype i.e. peripheral nervous system absence was rescued. The nervous system of embryos from the rescue crosses with *da^G32^*-GAL4 was visualised by immunohistochemistry using neuronal marker Futsch (*Drosophila* homologue to mammalian Microtubule associated protein 1B). Embryos homozygous for null mutant allele *da^10^* lack the entire PNS and have defects in CNS (Fig. 4B) compared to wt embryos (Fig. 4A). Expressing Da^wt^ in *da* null background rescued the neuronal phenotype as reported before (Giebel et al., 1997; Smith and Cronmiller, 2001) (Fig. 4C). In embryos expressing Da^D515G^ (Fig. 4D), Da^A614V^ (Fig. 4I), TCF4-A (Fig. 4J) or TCF4-B (Fig. 4K) in *da* null background the neuronal phenotype was rescued as well. Interestingly, Da^R578H^ that was unable to activate E-box controlled reporter expression *in vitro* (Fig. 2) and in the wing disc (Fig. 3D), rescued the development of the embryonic nervous system (Fig. 4E). Da^R580W^ (Fig. 4F), Da^R580L^ (Fig. 4G) and Da^R582P^ (Fig. 4H) were unable to rescue the neuronal phenotype of *da* null mutants, which is consistent with their inability to activate transcription from E-box.

Because rescue of *da* null mutants with expression of Da^wt^ using *da^G32^*-GAL4 driver failed, other GAL4 strains with broad expression like *tub*-GAL4, *69B*-GAL4 and *ubi*-GAL4 were used for rescue experiments. However, the rescue of *da* null embryonic lethality using these drivers was unsuccessful (data not shown). These results demonstrate that the exact dosage and spatial and temporal regulation of Da protein expression are highly important for *Drosophila* viability.

### Overexpression of Da, Da^PTHS^, TCF4-A, or TCF4-B by *GMR12B08*-GAL4 results in the rough eye phenotype

**Fig. 5.**
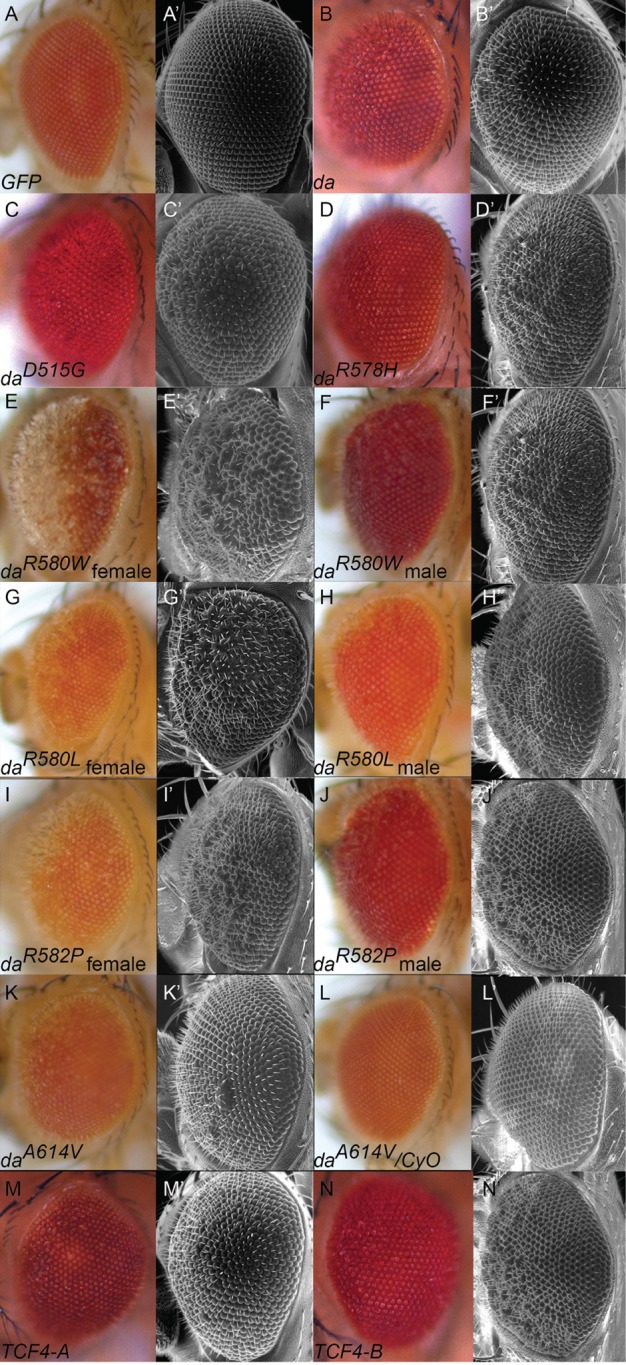
**Expression of Da^wt^, Da^PTHS^, TCF4-A and TCF4-B under neuronal driver *GMR12B08*-GAL4 results in rough eye phenotype.** Rough eye phenotype was observed when *GMR12B08*-GAL4 was homozygous. Overexpression of GFP served as a control showing no deviation from wild type eye (A,A′). Da^wt^ (B,B′), Da^D515G^ (C,C′), Da^R578H^ (D,D′), TCF4-A (M,M′) and TCF4-B (N,N′) caused rough eye phenotype with irregularly placed and occasionally fused ommatidia closer to the head midline half of the eye. Da^A614V^ caused similar phenotype only in double homozygous state (K,K′), one copy of *UAS-da^A614V^* was insufficient to induce the rough eye (L,L′). Da^R580W^ (E,E′), Da^R580L^ (G,G′) and Da^R582P^ (I,I′) resulted in eye pigmentation defects in addition to rough eye phenotype and the phenotype was enhanced in all females compared to males (F,F′,H,H′,J and J′).

Overexpression of Da^wt^, Da^PTHS^, TCF4-A or TCF4-B under ubiquitous *da^G32^-*GAL4 driver resulted in embryonic lethality. Overexpressing these transgenes under the control of the nervous-system specific *GMR12B08*-GAL4 resulted in viable flies with the rough eye phenotype (Fig. 5). During eye development, this driver line is weakly expressed in larval eye discs and strongly in larval optic lobes (Pfeiffer et al., 2008 and our unpublished results). Overexpression of Da^wt^ (Fig. 5B,B′), Da^D515G^ (Fig. 5C,C′), Da^R578H^ (Fig. 5D,D′), TCF4-A, (Fig. 5M,M′) or TCF4-B (Fig. 5N,N′) resulted in the rough eye phenotype only when *GMR12B08*-GAL4 driver was homozygous. Exceptionally, Da^A614V^ overexpression under *GMR12B08-*GAL4 led to the rough eye phenotype only in double homozygous state (Fig. 5K,K′,L,L′). *TCF4* and other *da* transgenes except *da^R580W^* remained heterozygous since double homozygous flies never survived into adulthood. Additionally, overexpression of Da^R580W^ (Fig. 5E,E′), Da^R580L^ (Fig. 5G,G′) and Da^R582P^ (Fig. 5I,I′) by the same driver resulted in partial loss of eye pigmentation. This phenotype was strongest in the case of Da^R580W^ female flies, with insertion of the transgene in the X-chromosome making them double homozygous. Interestingly, the rough eye phenotype was also weaker in the males of other transgenic lines compared to females carrying arginine mutations (R580L and R582P) with transgene insertions in the second chromosome (Fig. 5H,H′,J,J′). These results demonstrate high sensitivity of eye development to *da* transgene dosage. A614V mutation yielded the most subtle rough eye phenotype and all three arginine mutations R580W, R580L and R582P produced the strongest rough eye phenotype. Our results also showed that Da^R578H^, Da^D515G^, TCF4-A and TCF4-B behave similarly to Da^wt^ when overexpressed under *GMR12B08*-GAL4, indicating that all the mentioned proteins have transactivation activity during fruit fly eye development.

### Overexpression of Da, TCF4-B and Da^PTHS^ in young adult flies results in significantly altered survivorship

**Fig. 6.**
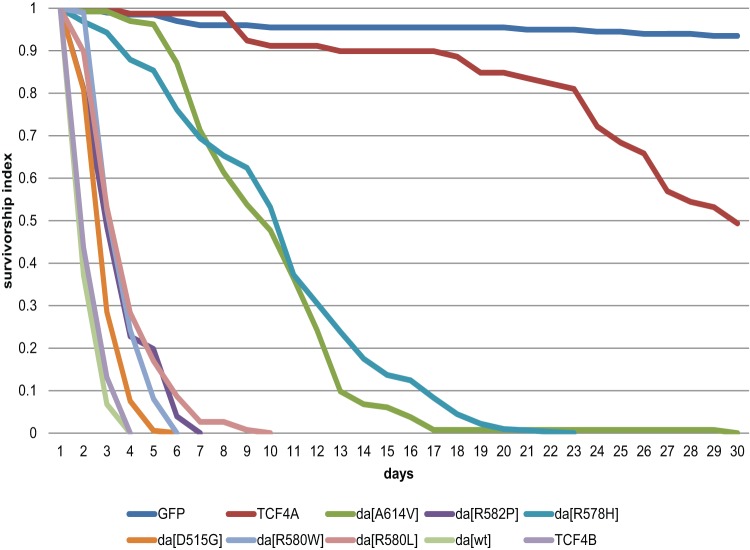
**Survivorship of adults after activation of Da^wt^, Da^PTHS^, or TCF4-B overexpression under *da^G32^*-GAL4 is significantly altered.** Using *tub*-GAL80^ts^ repressor during development allows activation of UAS-*da* transgenes expression under *da^G32^*-GAL4 only after eclosion of adults. The *x*-axis represents days from eclosion on day 0 to day 30, the *y*-axis represents survivorship index. Different UAS-transgenes are colour-coded. *tub*-GAL80^ts^/+; *da^G32^*-GAL4/UAS-*m*CD8-GFP flies were used as a control group.

Next, we wanted to evaluate the impact of Da in adult flies. For this the temperature sensitive repressor of GAL4, GAL80^ts^ was used. The overexpression of Da under the control of the ubiquitous *da^G32^*-GAL4 driver is lethal during embryogenesis (Giebel et al., 1997; Smith and Cronmiller, 2001). In order to overcome the lethality during development, we repressed GAL4 by GAL80^ts^. After the eclosion of adults, the collected virgin females were kept at restrictive temperature to inactivate GAL80^ts^. Activated overexpression of Da^wt^ or Da^D515G^ or *TCF4* long isoform B lead to lethality in 2-3 days (Fig. 6). The flies initially lost their flight ability and most of locomotor activity and died soon afterwards. Also, the activation of Da carrying PTHS-related arginine mutations unable to bind DNA (R580W, R580L, and R582P) lead to lethality in median 3-4 days (Fig. 6). Flies overexpressing the Da with weaker mutations, Da^R578H^ and Da^A614V^, resulted in median survival of 10-11 days while the control group flies overexpressing GFP survived generally 40 days (Fig. 6; Table S1). Surprisingly, the flies overexpressing the shorter TCF4-A isoform survived significantly longer than the flies overexpressing TCF4-B and were closer to the control group, with median survival of 30 days (Fig. 6; Table S1). All survivorship curves obtained were statistically significant by log-rank as compared to the GFP control curve (*P*<0.0001, Mantel–Cox test).

## DISCUSSION

In this study we show that Da, the only E-protein in *Drosophila* with highly conserved bHLH domain, functions as human TCF4 orthologue. As the overall identity of a protein sequence between *Drosophila* and mammals is usually around 40% between homologues and 80-90% within conserved functional domains (Pandey and Nichols, 2011), Da can be considered the orthologue for all three human E-proteins. In all experiments conducted in this study, TCF4 acted in very similarly as Da (Table 1), proving the possibility of modelling PTHS in the fruit fly. The two human TCF4 isoforms, TCF4-A and TCF4-B, were able to activate E-box dependent lacZ expression in *Drosophila*, and more importantly, to induce ectopic bristle formation in the adult thorax, to rescue embryonic nervous system development in *da* null embryos, and to induce the rough eye phenotype when overexpressed in the nervous system identically to Da. Altogether these results show that TCF4 has comparable activity in the fruit fly as Da.

**Table 1.**
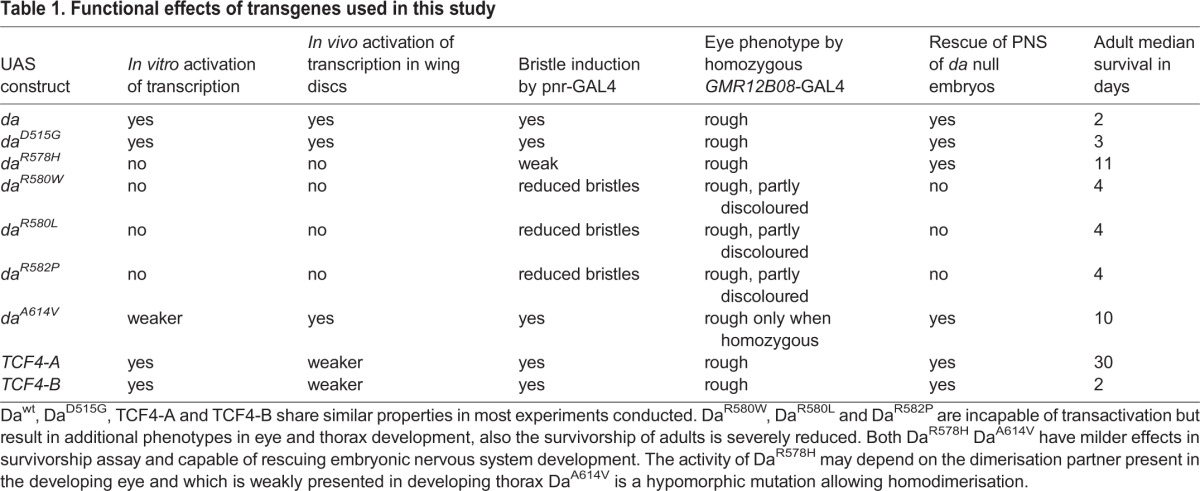
**Functional effects of transgenes used in this study**

To further study the PTHS-associated mutations in *Drosophila,* we introduced four mutations found in TCF4 (R580W, R578H, R582P and A614V) and two control mutations (D515G and R580L) into Da. We analysed the mutants by luciferase assay in a mammalian cell line in order to compare the results of Da directly to our previous results obtained with human TCF4 (Sepp et al., 2012). Subsequently we studied PTHS-associated Da mutants *in vivo* in E-box lacZ reporter assay, and in both rescue and overexpression experiments.

PTHS-associated arginine mutations R580W, R580L, and R582P abolished Da transactivation capability in luciferase reporter assays in HEK293 cells. Da^R580W^, Da^R580L^ and Da^R582P^ behaved similarly to each other in both overexpression and rescue experiments in the fruit fly. The rescue by *da^G32^*-GAL4 driver of *da* null embryonic nervous system phenotype failed when using Da proteins with these arginine mutations. When Da carrying one of above mentioned mutations was overexpressed in flies under the control of the nervous system specific driver *GMR12B08*-GAL4, the strongest eye phenotype was observed. These flies had rough and partially unpigmented eyes with fused ommatidia consistent with Da having an important role in *Drosophila* eye development (Brown et al., 1996). In addition, overexpression of these arginine mutants under *pnr*-GAL4 caused malformation of the thorax. Altogether, these results indicate that mutations R580W, R580L and R582P abolish the Da transactivation capability resulting in dominant-negative effects. This is in line with the previous data about the corresponding mutations in TCF4 having dominant-negative effects *in vitro* (Sepp et al., 2012).

R578H differed from the other three arginine mutations (R580W, R580L and R582P) in *in vivo* experiments. Although Da^R578H^ was unable to activate reporter gene expression in luciferase assay carried out in mammalian cell line HEK293 and in lacZ assay *in vivo*, it caused rough eye phenotype similar to Da^wt^ when overexpressed by *GMR12B08*-GAL4. Furthermore, Da^R578H^ rescued *da* null embryonic neuronal phenotype when expressed using *da^G32^*-GAL4. Also Da^R578H^ showed weak induction of ectopic bristles. Taken together these results indicate that transactivation capability of Da^R578H^ probably depends on its dimerisation partners, which could be lacking in mammalian cell line and weakly presented in the wing disc notum. Similarly, we have previously found that while TCF4 carrying the R578H mutation is unable to bind to E-box *in vitro* as a homodimer or in complex with either ASCL1 or NEUROD2, it does not act in dominant negative manner in reporter assays in mammalian cells (Sepp et al., 2012).

The A614V mutation positioned in the second helix of the bHLH domain showed the mildest effects. Da^A614V^ was able to activate E-box-specific transcription *in vitro* and *in vivo*. Expressing Da^A614V^ using *da^G32^*-GAL4 rescued *da* null embryonic neuronal phenotype. Overexpression using *GMR12B08*-GAL4 resulted in the rough eye phenotype only when both of the transgenes were homozygous, indicating that this mutation causes hypomorphic effects. This is consistent with our recent study which showed that the A614V mutation leads to lower levels of TCF4 because of reduced protein stability (Sepp et al., 2012).

The control mutation generated by us, D515G, did not reduce Da transactivation capability *in vitro* and behaved similarly to Da^wt^ *in vivo*. This shows that D515 positioned outside of the conserved bHLH is not required for Da transcriptional activity. The other control mutation generated by us, R580L, where the same arginine was mutated as in Da^R580W^, led to dominant-negative effects *in vivo* similarly to R580W. At least in the case of R580, the mutation specificity, whether it was mutated into tryptophan or leucine, made no difference in our study.

In rescue experiments with tested driver strains (*69B*-GAL4, *tub*-GAL4, *ubi*-GAL4, *GMR12B08*-GAL4, *da^G32^*-GAL4) all Da transgenes failed to rescue *da* null embryonic lethality. Apparently the successful rescue of *da* null lethality closely mimics the endogenous Da expression. *da^G32^*-GAL4, comprising of 3.2 kb of *da* gene covering the promoter, the first intron, and the upstream noncoding region (Wodarz et al., 1995), is widely used as a ubiquitous driver line. Most probably the expression of this driver line is far too strong compared to the native expression of *da* gene as Da has been shown to positively autoregulate its own expression via a transcriptional feedback loop (Bhattacharya and Baker, 2011; Smith and Cronmiller, 2001). If *da^G32^-*GAL4 expression is regulated by Da itself, then Da overexpression might drive even stronger GAL4 expression, resulting in a positive feedback loop. Furthermore, it has been hypothesised that *da^G32^-*GAL4 lacks putative regulatory repressor elements since using a 15 kb genomic *da* transgene that has an additional 12 kb of downstream sequence rescues *da* null embryonic lethality (Smith and Cronmiller, 2001).

Little is known about the role of E proteins in adult nervous system. Here we show that exact temporal and spatial expression of Da/TCF4 remains vitally important during adulthood of fruit flies. We show that overexpression of Da/TCF4 in adults leads to lethality within 2-3 days. Surprisingly, TCF4 isoforms A and B lead to strikingly different outcomes when overexpressed in adult fruit flies. While the long isoform TCF4-B behaved identically to Da, TCF4-A affected the survival only slightly compared to the control group. This could be related to the lack of interaction capability of much shorter N terminus of isoform A in fruit fly or different regulation of subcellular location and dimerisation of the alternative TCF4 isoforms (Sepp et al., 2011). Analysis of survival divided the PTHS related mutations into severe (R580W, R580L and R582P) and milder (R578H and A614V) according to survivorship. The severe mutants led to lethality within 3-4 days and the milder ones in 10-11 days. Further experiments with cell type or tissue-specific drivers would help to understand the role of E-proteins during adulthood in more details.

The fact that overexpression of wild type as well as dominant negative forms of Da causes comparable reduction in survival and induction of the rough eye phenotype raises the possibility that overexpression of wt protein is also eliciting dominant negative effects as suggested earlier (Sweatt, 2013). One explanation for this phenomenon could be that excess homodimers outcompete transcriptionally more potent heterodimers at various promoter sites. Intriguingly, recent studies suggest that in addition to TCF4 haploinsufficiency, increased TCF4 dose is also a risk factor for disturbed cognitive development as a TCF4 duplication has been described in a patient with developmental delay (Talkowski et al., 2012) and a partial duplication in a patient with major depressive disorder (Ye et al., 2012). Nevertheless, in case of induction of ectopic bristles we observed opposite effects for Da^wt^ and dominant negative Da mutants, indicating that in addition to its dominant negative effects, excess wt protein also has specific effects during development.

In patients with PTHS just one copy of *TCF4* is mutated or deleted. Seemingly the most relevant way to model PTHS in animal models would be to use the appropriate heterozygotes of the orthologous protein. However, in *Drosophila* there is a sole E-protein Da corresponding to all three mammalian E-proteins. In a way the heterozygous Da null mutation corresponds to the heterozygous deletion of all three E-proteins in mammals. Accordingly, Da as the only binding partner of class II bHLH proteins has a large variety of roles outside nervous system. As TCF4 is highly expressed in the nervous system we have chosen here the approach to overexpress the mutated alleles specifically in the nervous system in a wild type background. Overexpression of Da^PTHS^ under the nervous system specific *GMR12B08*-GAL4 led to viable flies and we were able to create stocks with each mutation generated in this study. An alternative tactic to model PTHS and to mimic dosage loss by TCF4 deletions would be to slightly downregulate Da expression nervous system specifically by RNAi. Additional studies are needed to generate and compare different PTHS models and to perform behavioural tests that would give valuable information about cognition and social behaviour of the PTHS model flies.

In conclusion, this study is the first where experiments with PTHS-associated mutations have been performed *in vivo*. We have verified Da as a functional TCF4 homologue, described similarities between Da and TCF4 carrying the same mutations, and obtained insights how PTHS-associated mutated Da genes could affect *Drosophila* embryonic nervous system development and thoracic bristle formation. The similarities between the effects of PTHS-associated mutations on Da and TCF4, ranging from hypomorphic to dominant-negative, prove that these proteins have similar functions and Da can be used for modelling of PTHS in *Drosophila melanogaster*. Our novel models of PTHS in *Drosophila* allow the design of further studies addressing the molecular mechanisms and treatment of PTHS.

## MATERIALS AND METHODS

### *Drosophila* stocks

All *Drosophila* stocks and crosses were kept on malt and semolina based food with 12 h light and dark daily rhythms at 25°C with 60% humidity unless otherwise noted. *Drosophila* strains used in this study were *da^G32^-*GAL4 (Wodarz et al., 1995), *69B-*GAL4 (Brand and Perrimon, 1993), and *tub-*GAL4 provided by Riitta Lindström, *ubi*-GAL4 provided by Mari Teesalu, *EE4-lacZ;pnr-GAL4/T(2,3)SM6-TM6B* kindly provided by Christos Delidakis, *GMR12B08*-GAL4 (Pfeiffer et al., 2008), *da^10^,FRT40A* (BL#5531), *UAS-da^G^* (BL#37291), *UAS-mCD8-GFP* (BL#5137), and *tub-*GAL80^ts^ (BL#7019) from Bloomington Stock Center at Indiana University, USA. The following transgenic lines were generated in this study: UAS-*da^D515G^*, UAS-*da^R578H^*, UAS-*da^R580W^*, UAS-*da^R580L^*, UAS-*da^R582P^*, UAS-*da^A614V^*, UAS-*TCF4-B*, and UAS-*TCF4-A*.

### Mutagenesis, cloning and transgenesis

The amino acid sequences were aligned and homology of human E-proteins and Da was calculated using Clustal Omega 2.1 (EMBL-EBI, Cambridge, UK). Site-directed mutagenesis was performed using the partial *da* cDNA construct GH10651-pOT_2_ as a template (Drosophila Genomics Resource Center, Bloomington, IN, USA). Primers were designed with that *de novo* restriction sites created next to the mutation with no change in amino acid sequence. The primer sequences are listed in Table S2. The constructs obtained by PCR were sequenced and subcloned into full-length *da* cDNA construct LP14713-pOT_2_ (Drosophila Genomics Resource Center) using SphI and NruI restriction sites. Full-length *da* carrying appropriate PTHS-associated mutation together with TCF4-A(-) and TCF4-B(-) cDNAs from the respective pCDNA3.1 vectors (Sepp et al., 2011) were then subcloned into pUAST vector. Generation of transgenic flies with random insertions was ordered from Fly Facility (Clermont-Ferrand Cedex, France).

### DNA transfection and luciferase assay

The transfection and luciferase assay was performed as described before (Sepp et al., 2011). Briefly, HEK293 cells obtained from ATCC (LGC Standards GmbH, Wesel, Germany) and routinely tested for contamination were transfected using LipoD293™ (SignaGen Laboratories, Gaithersburg, MD, USA) with pCDNA3.1 based *TCF4* or *da* constructs and firefly luciferase construct pGL4.29[luc2P/12µE5/Hygro] or pGL4.29[luc2P/min/Hygro] and *Renilla* luciferase construct pGL4.29[hRlucP/min/Hygro] for normalisation. Transfections were carried out as duplicates on a 48-well plate. After 24 h cells were lysed with 50 µl Passive Lysis Buffer (Promega, Madison, Wisconsin, USA). Dual-Glo Luciferase assay (Promega) was performed following manufacturer's protocol and luminescence was measured with GENios Pro Multifunction Microplate Reader (Tecan Group, Männedorf, Switzerland). For data analysis, background signals from untransfected cells were subtracted and firefly luciferase signals were normalised to *Renilla* luciferase signals. The data was then log-transformed, auto-scaled, means and standard deviations were calculated and Student *t*-tests were performed. The data was back-transformed for graphical representation.

### *In vivo* lacZ reporter assay

For expression of transgene in wing discs, ubiquitous temperature-sensitive *tub*-GAL80^ts^ repressor was used to avoid larval lethality caused by *pnr*-GAL4>UAS-*da*. Vials with 3rd instar larvae were transferred from permissive temperature at 18°C to restrictive temperature at 30°C for 24 h before dissection of wing discs and afterwards moved to 25°C until adults emerged. Each *da* mutant strain under UAS was crossed to *EE4-lacZ; pnr-GAL4/T(2,3)SM6-TM6B*. 3rd instar wing discs of their progeny were dissected and the X-gal staining was performed. Expression of transgene in wing discs and X-gal (5-bromo-4-chloro-3-indolyl-β-D-galactopyranoside) histochemistry was performed as described before (Zarifi et al., 2012). For imaging, Olympus BX61 microscope (Tokyo, Japan) with UPlanSApo 20×/0.75 objective was used.

### RNA isolation and RT-PCR

Total RNA was isolated from embryos using RNeasy Micro Kit (Qiagen, Hilden, Germany) and treated with TURBO DNase (Ambion, Thermo Fisher Scientific, Waltham, MA, USA). First strand cDNA was reverse transcribed from 1 µg of RNA using oligo(dT)_20_ primer and Superscript III Reverse Transcriptase (Invitrogen, Thermo Fisher Scientific, Waltham, MA, USA). PCR was performed using FirePol DNA polymerase (Solis Biodyne, Tartu, Estonia). Primer annealing temperature was 55°C and primer sequences are presented in Table S2. After PCR, restriction analysis was performed using restriction sites created during mutagenesis (Fig. S1).

### Immunohistochemical staining of embryos

The following primary antibodies and dilutions were used: rabbit EGFP antiserum (provided by Andres Merits, Tartu University), 1:2000; mouse monoclonal 22C10 anti-Futsch antibody (deposited by Benzer, Seymour/Colley, Nansi, obtained from the Developmental Studies Hybridoma Bank, University of Iowa, IA, USA), 1:20. The following secondary antibodies and dilutions were used: goat anti-mouse Alexa 594, 1:1000; and goat anti-rabbit Alexa 488 (both ImmunoResearch Laboratories, Inc., West Grove, PA, USA), 1:1000. *Drosophila* embryos were dechorionated using 2% sodium hypochlorite (Sigma-Aldrich, St. Louis, MO, USA) and fixed using 4% formaldehyde (AppliChem GmbH, Darmstadt, Germany) in PEM buffer (100 mM PIPES, 1 mM EGTA, 2 mM MgSO_4_, pH 7.4) and stored in methanol at −20°C until used. Primary antibody labelling was performed overnight on overhead rotator at 4°C. Secondary antibodies were preadsorbed to wt embryos before use. Incubation with secondary antibodies was performed for 3 h on overhead rotator at room temperature. All staining procedures and washes were performed with 0.1% PBS-Triton X-100. Fluorescently labelled embryos were mounted in Vectashield mounting medium with DAPI (Vector Laboratories, Burlingame, CA, USA). For image collection, Zeiss LSM 510 Meta confocal microscope with Pln Apo 20×/0.8 DICII objective (Carl Zeiss Microscopy GmbH, Jena, Germany) was used. Suitable layers were selected using Zeiss LSM Image Browser (Carl Zeiss Microscopy). Homozygous *da^10^* embryos were selected by the lack of GFP marker expression present in balancer chromosome.

### Light microscopy and imaging of adult flies

Flies were euthanised using chloroform (Sigma-Aldrich). For capturing eye and thorax images, Zeiss Stereo Discovery V8 microscope and Zeiss Axiocam MRc camera were used. For scanning electron microscopy, Zeiss Evo LS15 (Carl Zeiss Microscopy) was used.

### Evaluating the lifespan of *da* transgenic flies

*w^−^; tub-*GAL80^ts^; *da^G32^*-GAL4 flies were crossed to *da* transgenic flies with insertions in second chromosome (lines UAS-*da^D515G1^*, UAS-*da^R578H3^*, UAS-*da^R580L4^*, UAS-*da^R582P2^*, UAS-*da^A614V3^*, UAS-*TCF4-B^4^*) or in third chromosome (lines UAS-*da^R580W3^*, UAS-*da^G^*, UAS-*mCD8-*GFP, UAS-*TCF4-A^2^*) and raised in permissive temperature 18°C where GAL80^ts^ represses the activity of GAL4. The progeny – eclosed virgin females – were collected and transferred to restrictive temperature 30°C where GAL80^ts^ is inactive and GAL4 is produced. Altogether, on average 200 flies (from at least 79 up to 314) were collected from each cross, maintained in uncrowded vials, counted on daily bases, and changed to new vials at three-day intervals. Survivorship index was calculated by dividing the survivors with the starting number of flies. Comparison of survival curves and *P*-value calculations were performed using Mantel–Cox log rank method with Prism 6.0 GraphPad Software (La Jolla, CA, USA).
